# Stimulus-responsive proteins involved in multi-process regulation of storage substance accumulation during rice grain filling under elevated temperature

**DOI:** 10.1186/s12870-023-04563-7

**Published:** 2023-11-08

**Authors:** Yufei Zhao, Tongyang Yin, Xuan Ran, Wenzhe Liu, Yingying Shen, Hao Guo, Yuxuan Peng, Chen Zhang, Yanfeng Ding, She Tang

**Affiliations:** 1 College of Agronomy, Nanjing Agricultural University, 210095 Nanjing, People’s Republic of China; 2 Jiangsu Collaborative Innovation Center for Modern Crop Production, 210095 Nanjing, People’s Republic of China

**Keywords:** Rice, Elevated temperature, Quality, Proteomics, Responsive to stimulus, Grain filling

## Abstract

**Background:**

The intensified global warming during grain filling deteriorated rice quality, in particular increasing the frequency of chalky grains which markedly impact market value. The formation of rice quality is a complex process influenced by multiple genes, proteins and physiological metabolic processes. Proteins responsive to stimulus can adjust the ability of plants to respond to unfavorable environments, which may be an important protein involved in the regulation of quality formation under elevated temperature. However, relatively few studies have hindered our further understanding of rice quality formation under elevated temperature.

**Results:**

We conducted the actual field elevated temperature experiment and performed proteomic analysis of rice grains at the early stage of grain filling. Starting with the response to stimulus in GO annotation, 22 key proteins responsive to stimulus were identified in the regulation of grain filling and response to elevated temperature. Among the proteins responsive to stimulus, during grain filling, an increased abundance of signal transduction and other stress response proteins, a decreased abundance of reactive oxygen species-related proteins, and an increased accumulation of storage substance metabolism proteins consistently contributed to grain filling. However, the abundance of probable indole-3-acetic acid-amido synthetase GH3.4, probable indole-3-acetic acid-amido synthetase GH3.8 and CBL-interacting protein kinase 9 belonged to signal transduction were inhibited under elevated temperature. In the reactive oxygen species-related protein, elevated temperature increased the accumulation of cationic peroxidase SPC4 and persulfide dioxygenase ETHE1 homolog to maintain normal physiological homeostasis. The increased abundance of alpha-amylase isozyme 3E and seed allergy protein RA5 was related to the storage substance metabolism, which regulated starch and protein accumulation under elevated temperature.

**Conclusion:**

Auxin synthesis and calcium signal associated with signal transduction, other stress responses, protein transport and modification, and reactive oxygen species-related proteins may be key proteins responsive to stimulus in response to elevated temperature. Alpha-amylase isozyme 3E and seed allergy protein RA5 may be the key proteins to regulate grain storage substance accumulation and further influence quality under elevated temperature. This study enriched the regulatory factors involved in the response to elevated temperature and provided a new idea for a better understanding of grain response to temperature.

**Supplementary Information:**

The online version contains supplementary material available at 10.1186/s12870-023-04563-7.

## Background

The global mean surface temperature was predicted to increase by 1.0 °C to 5.7 °C by 2100 compared to 1850–1900 [1]. The temperature rise in China was faster than the global average temperature in the same period. In recent years, the occurrence of high temperature events in China remarkably increased due to the effect of climate change [2–4]. As one of the major rice-growing countries, China contributed more than 30% of global rice production [5]. However, with the increased frequency and magnitude of high temperature in the rice growing season, rice cultivation had encountered great challenges [6].

The temperature rise will impose stressful influences on different growth and developmental stages throughout the rice growing season [7]. However, elevated temperature has a greater effect on the reproductive stage, especially in the grain-filling stage [8]. From the biological point of view, grain filling refers that the zygote develops into an embryo through cell division and differentiation and the polar nucleus develops into endosperm by photosynthetic assimilation deposition and storage protein synthesis [9]. An et al. [10] integrated the developmental features of both embryo and endosperm and proposed stages of developmental grain: stage 1: embryo morphogenesis (0–10d after flowering); stage 2: endosperm filling (10–30d after flowering); stage 3: seed maturation (30d after flowering-maturity). At maturity, starchy endosperm accounts for 89%–91% of rice caryopsis [11]. Therefore, the storage substances deposition into the endosperm cells during grain filling is a critical stage in grain development which determine rice yield and quality [12–15]. The peak of grain substance accumulation occurs between 10d—20d after flowering and the grain weight is almost unchanged during 20d-30d after flowering [10, 16]. Grain substance accumulation depends not only on genetic background but also on environmental conditions [17]. High temperature in rice grain-filling stage shorted starch-filling duration and increased starch degradation to lead grain phenotype with less translucence [18–22]. Increased temperature also reduced amylose content, changed amylopectin content, and increased protein accumulation to further change rice cooking and nutritional quality [23–27]. However, the molecular mechanism by which elevated temperature changes the physicochemical properties of rice storage substances and quality formation has not been elucidated. Hence, the lack of molecular mechanism pushes researchers to explore insight into the responses of rice grain to high temperature [24, 28, 29]. Our previous studies have clarified the physiological effects of temperature on rice grain [30, 31]. Therefore, we hope to further investigate the molecular mechanism of increased temperature regulation on grain substance accumulation and rice quality based on the previous study results.

Genomics, transcriptomics, proteomics and metabolomics provide large-scale analysis of genes, transcripts, proteins and metabolites to revolutionize plant science studies [32]. Proteins play an indispensable role in almost every cellular function [33–36]. Therefore, proteomics analysis possesses an irreplaceable advantage which is more important than other levels [37]. Abundant studies have been performed on rice proteomics which can be divided into three major categories: nutrition component, growth and development, and stress responses [38]. Plants are inevitably affected by environmental factors including drought, salinity and extreme temperatures, etc. during growth and development. A number of proteomic studies have been carried out on stress response [39–42]. The rice proteomic studies under high temperature have mainly focused on the mechanism of rice response, and some researchers have identified some pathways of rice response to high temperature. Timabud et al. [43] found considerable proteins involved in redox homeostasis and carbohydrate biosynthetic pathways were important to rice grains in response to high temperature. Liao et al. [44] conducted a proteomic analysis with different heat-tolerant rice lines to elucidate the differential abundance proteins related to the Calvin cycle, the glycolytic pathway, the tricarboxylic acid cycle which regulated rice grain substance accumulation under high temperature. The identification of the proteins which are responsive to elevated temperature is vital to understanding the molecular mechanisms of temperature regulation effects. Numerous studies also inspired researchers that some stress-related proteins were pivotal in rice grain development, such as heat shock proteins (HSP), peroxidases and lipid transfer proteins [45–47]. Kumar et al. [48] revealed that 72% of the identified differential proteins belonged to defense and stress responses under elevated temperature by two-dimensional electrophoresis (2-DE) and MALDI-TOF/MS based proteomics approaches. Although several genes and proteins have been identified that were involved in the response to high temperature stress, much research remains to be done to fully understand the regulatory networks of stress-responsive proteins [24, 49, 50].

Since 2012, we continuously conducted actual field warming treatments by the Free-Air Temperature Enhancement (FATE) facility. In our previous study, we have found the changes in the physicochemical properties of starch, and the imbalance of storage protein may cause deteriorated rice quality [30, 31, 51–53]. A deeper understanding of the internal regulatory network is essential to explain the rice quality formation under warming. A number of studies have shown that stress proteins can adjust the ability of plants to respond to unfavorable environments. However, the lack of research content limits our understanding of stress proteins that regulate grain substance accumulation in rice under elevated temperature. Therefore, we performed proteomic analysis of rice grains at the early stage of grain filling under natural and elevated temperature. Our results identified some candidate proteins responsive to stimulus and revealed these proteins on the regulatory pathway for grain substance accumulation under elevated temperature, which enriches the protein regulatory network for grain quality formation under elevated temperature.

## Results

### Elevated temperature affected the grain filling and deteriorated rice quality

**Fig. 1:**
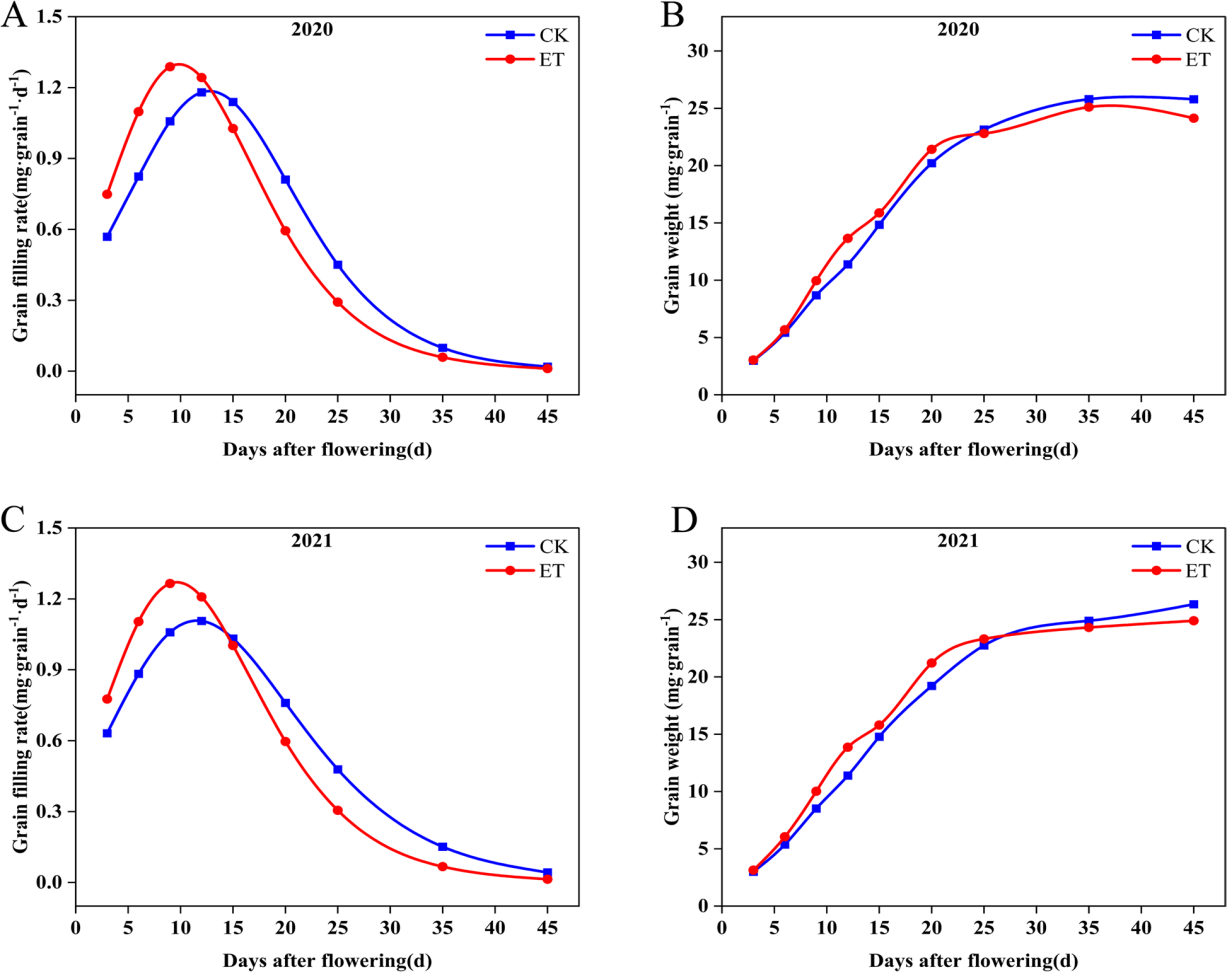
Effects of elevated temperature on grain filling. **A**, **C** Grain filling rate was in 2020 and 2021 under elevated temperature, respectively. **B**, **D** Grain weight was in 2020 and 2021, respectively. Note: *CK* Natural temperature, *ET* Elevated temperature

Figure 1 showed grain weight dynamics and grain filling rates of W3 at natural temperature and elevated temperature for two years. The effect of temperature on grain weight was consistent for two years. Elevated temperature increased grain weight before 25d after flowering. Subsequently, the up-regulated trend of grain weight disappeared and presented an opposite trend under elevated temperature. The effects of warming on the grain filling rate were consistent with the grain weight. Elevated temperature accelerated the grain filling in the early filling stage (3d to 12d after flowering). However, in the middle and late filling stages, insufficient filling stamina under elevated temperature was shown. Ultimately, the active grain-filling period was shortened under elevated temperature (Table S1). The effect of temperature on grain filling process ultimately reduced the seed setting rate and grain weight which led to a decrease in the theoretical yield (Table S2).

**Fig. 2:**
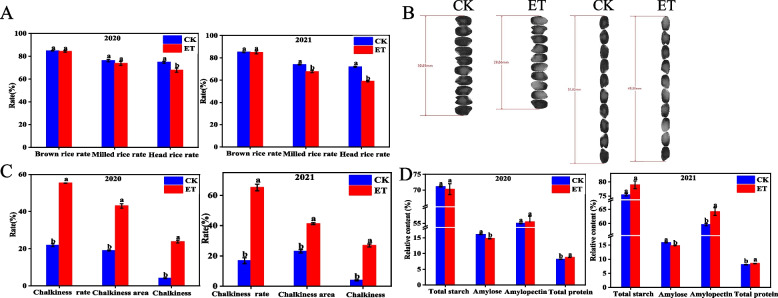
Effects of elevated temperature on rice quality. **A** Milling quality; **B** Scan imagery on rice grain length and width; **C** Chalkiness characteristic; **D** The content of starch components and total protein. Note: CK: natural temperature; ET: elevated temperature. Bars are the stand deviation. Values followed by different letters are significantly different at 5% probability

The effect of elevated temperature on rice quality was shown in Fig. 2. There was no significant influence on brown rice rate and milled rice rate under elevated temperature in 2020, and the head rice rate decreased significantly by 9.29%. However, in 2021, elevated temperature resulted in a decrease in milled rice rate at the significance level. Similarly, the head rice decreased significantly by 17.90% in 2021. The length of grain significantly reduced in 2020 and 2021, whereas the width did not change significantly in 2021, only a decreasing trend. However, the length/width was not affected under elevated temperature (Table S3). Chalkiness was the most sensitive appearance trait and changed significantly under elevated temperature. The two-year results consistently showed that increased temperature deteriorated chalky characteristics, especially the chalkiness, which increased by 527.36% and 596.89% in 2020 and 2021, respectively (Fig. 2).

The effect of temperature on filling ultimately also led to change in the two main storage substances, starch and storage protein. Elevated temperature had no significant effect on the total starch content, but significantly reduced the amylose content by 7.99% and 6.84% in 2020 and 2021, respectively. Elevated temperature significantly increased the content of amylopectin only in 2021. Elevated temperature had no significant influence on total starch, and led to an increase in total protein content, especially in 2020 which increased by 7.12%.

Under elevated temperature, the changes in Rapid viscosity analyzer (RVA) profile characteristics in 2020 and 2021 were consistent (Table 1). Elevated temperature had a positive effect on peak viscosity, breakdown viscosity and past temperature. Among them, compared with natural temperature, the breakdown viscosity increased by 49.56% and 72.87% in 2020 and 2021, respectively. Hot paste viscosity, final viscosity, setback viscosity and past time decreased significantly. Compared to 2020, the decline was even greater in 2021, especially the setback viscosity, which dropped by 363.27%.

**Table 1:** Effects of elevated temperature on RVA profile characteristics

Year	Treatment	Peak viscosity /cP	Hot paste viscosity /cP	Breakdown viscosity /cP	Final viscosity /cP	Setback viscosity /cP	Past time /min	Past temperature /℃
2020	CK	2694.50b	2073.00a	621.50b	3115.50a	421.00a	6.87a	73.65b
	ET	2923.50a	1994.00b	929.50a	2860.00b	-63.50b	6.47b	75.95a
2021	CK	2989.67b	2277.00a	712.67b	3185.67a	196.00a	6.82a	75.17b
	ET	3321.00a	2089.00b	1232.00a	2805.00b	-516.00b	6.25b	78.57a

Note: *CK* Natural temperature, *ET* Elevated temperature. Different letters indicate significant differences (*P* < 0.05)

The quality of the cooking and eating was measured by taste meter and hardness viscometer (Table 2). The appearance and taste of rice significantly decreased under elevated temperature, especially in 2020. Elevated temperature had a negative impact on rice viscosity, balance and elasticity. The overall score of rice under elevated temperature decreased by 4.63% and 3.25% in 2020 and 2021, respectively.

**Table 2:** Effects of elevated temperature on the eating quality of rice

Year	Treatment	Appearance	Hardness	Viscosity	Balance	Elasticity	Taste	Overall score
2020	CK	8.7a	5.30b	0.74a	0.17a	0.75a	8.47a	77.77a
	ET	8.1b	5.78a	0.68a	0.13a	0.73a	8.03b	74.17b
2021	CK	9.60a	4.01b	0.73a	0.18a	0.76a	9.30a	85.17a
	ET	8.97b	4.89a	0.63a	0.15a	0.70b	8.87b	82.40b

Note: *CK* Natural temperature, *ET* Elevated temperature. Different letters indicate significant differences (*P* < 0.05)

### Proteins responsive to stimulus in coping with elevated temperature

We attempted to further investigate the effect of temperature on grain storage substance accumulation and quality formation by proteomics. Based on the DIA (Data Independent Acquisition) experiment, a total of 22,930 unique peptides and 6650 proteins were identified in grains at 3, 6, 9, 12 and 15d after flowering under natural temperature and elevated temperature (Fig. S1A, B). Fig. S1C indicated most protein coverage was < 30%. In addition, PCA (Principal Component Analysis) 1 and PCA2 accounted for 48.64% and 11.44% of the variability, which indicated good sample reproducibility for proteomics (Fig. S1D).

**Fig. 3:**
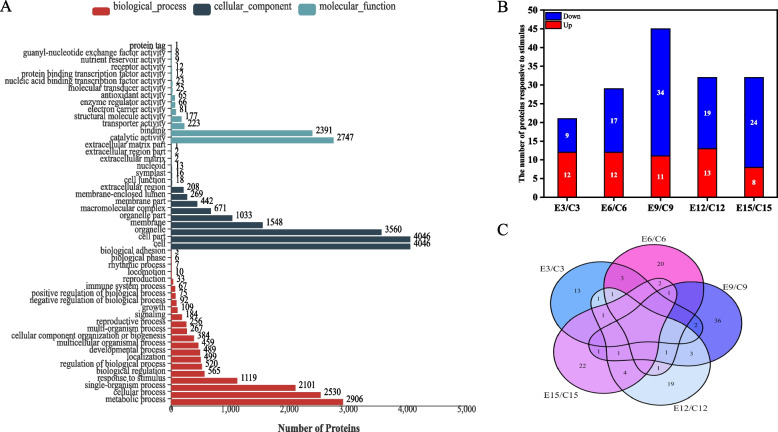
Changes of differential proteins under elevated temperature. **A** GO annotation statistics of differentially expressed proteins during grain substance accumulation and under elevated temperature; **B** Changes in the number of differential proteins under warming on different days after flowering; **C** Venn diagram analysis of differential proteins in response to elevated temperature on different days after flowering. Note: E3/C3 represents differentially responsive to stimulus proteins at 3d after flowering under elevated temperature. Other representations are similar

Figure 3A showed the identified differentially expressed proteins were mainly divided into molecular function, cellular components and biological processes by GO (Gene Ontology) analysis. ​Among them, the identified differential proteins were mostly involved in the biological processes, and more than 1000 proteins were enriched in response to stimulus, single-organism process, cellular process and metabolic process which belonged to biological processes. we selected 1119 proteins that belonged to responsive to stimulus for further analysis.

**Fig. 4:**
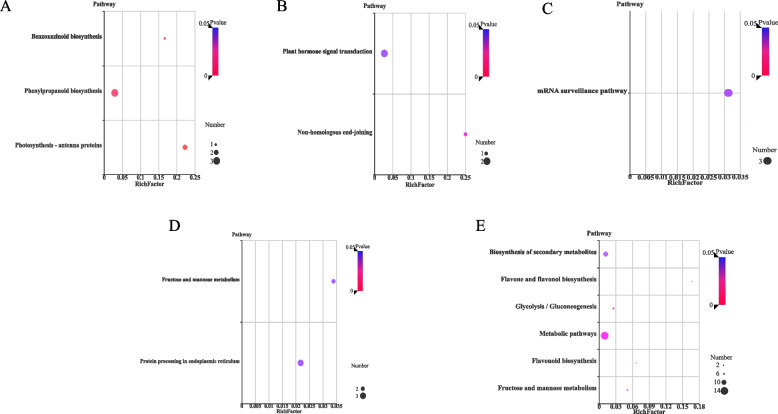
KEGG-enriched bubble plots of differential proteins under elevated temperature at different days after flowering. **A** 3d after flowering; **B** 6d after flowering; **C** 9d after flowering; **D** 12d after flowering; **E** 15d after flowering

Figure 3B showed the number of differential proteins under elevated temperature (Fold change >  = 2; *P* value < 0.05). ​At 9d after flowering, the highest number of differential proteins was found under elevated temperatures, including 11 up-regulated differential proteins and 34 down-regulated differential proteins. The number of total differential proteins was equal at 12d and 15d after flowering, but the number of down-regulated differential proteins at 15d after flowering were more than that at 12d. A Venn diagram analysis was conducted to determine the dynamics of differential proteins under elevated temperature at different days after flowering (Fig. 3C). We further analyzed the KEGG (Kyoto Encyclopedia of Genes and Genomes)-enriched bubble plot of differential proteins under elevated temperature (Fig. 4). In the KEGG-enriched bubble plots, differential proteins responsive to stimulus were more enriched at 12d and 15d after flowering, especially at 15d after flowering. Protein processing in endoplasmic reticulum (folding, classification and degradation), fructose and mannose metabolism (carbohydrate metabolism) were significantly enriched at 12d after flowering. At 15d after flowering, it was significantly enriched in fructose and mannose metabolism (carbohydrate metabolism), flavonoid biosynthesis (biosynthesis of other secondary metabolites), glycolysis/gluconeogenesis (carbohydrate metabolism), metabolic pathways and biosynthesis of secondary metabolites. Therefore, differential proteins responded to elevated temperature by different metabolic pathways at different days after flowering.

The differential proteins under elevated temperature can be divided into seven types according to GO categories (Table S4). The differential proteins under elevated temperature were diversified, among which proteins involved in stress response, chemical stress and abiotic stress were the most, accounting for 26.51%, 17.87% and 17.58%, respectively.

### Proteins responsive to stimulus during grain substance accumulation

**Fig. 5:**
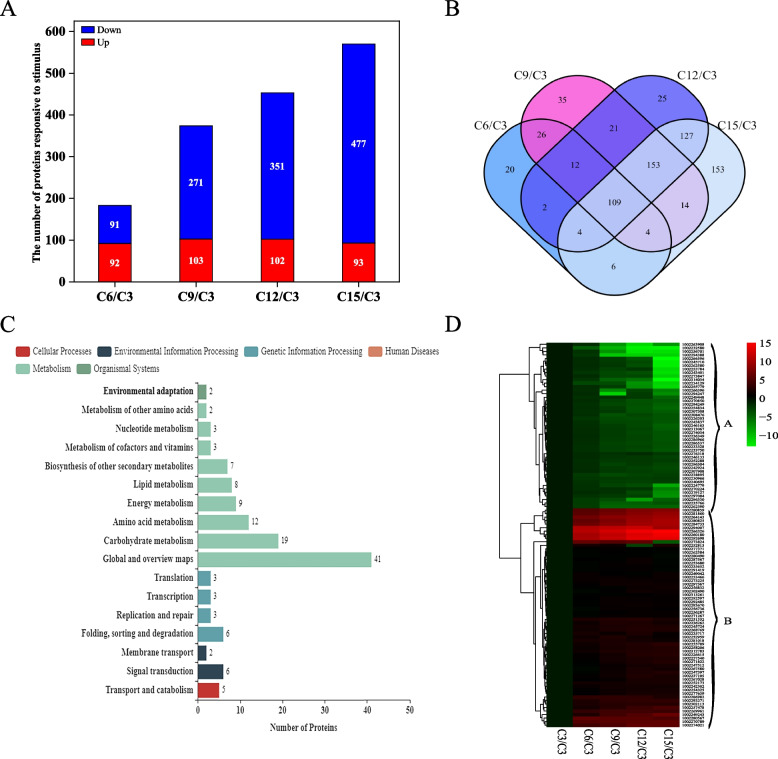
The number, metabolic pathways and classification of differential proteins during grain substance accumulation. **A** The number of differential proteins during grain substance accumulation; **B** 109 proteins were consistently differentially expressed during grain substance accumulation by venn diagram analysis; **C** KEGG metabolic pathway analysis of 109 proteins that are consistently differentially expressed during grain substance accumulation; **D** Cluster analysis of expression patterns of 109 differential proteins. Red indicates the abundance of protein was up-regulated (the log2 relative expression level was more than 0), and green indicates the abundance of protein was down-regulated (the log2 relative expression level was less than 0). The down-regulated expression of differential proteins was classified as A class, and the up-regulated expression was classified as B class. Note: C6/C3 represents significantly differential proteins at 6d after flowering compared to 3d after flowering. Other representations are similar

After studying the proteins responsive to stimulus under elevated temperature, we further investigated the role of proteins in grain filling. Figure 5A showed the number of differential proteins which were at 6, 9, 12 and 15d after flowering compared with 3d after flowering (Fold change >  = 2; *P* value < 0.05). The total differential proteins gradually increased as the grain substance accumulation progressed. There was no significant difference in the number of up-regulated differential proteins. However, the number of down-regulated differential proteins gradually increased with the progress of grain substance accumulation. Figure 5B indicated a total of 109 differential proteins which were significantly up- or down-regulated at 6, 9, 12 and 15d after flowering (Table S6). The 109 differential proteins were mainly involved in carbohydrate metabolism, amino acid metabolism, energy metabolism and lipid metabolism and other metabolic processes which were necessary for grain substance accumulation by KEGG metabolic pathway analysis (Fig. 5C).

To further clarify the role of differential proteins in grain filling, the expression patterns of 109 differential proteins were distinguished by hierarchical clustering analysis (Fig. 5D). With the grain substance accumulation, the down-regulated expression of differential proteins was classified as A class, and the up-regulated expression of differential proteins was classified as B class. The differential proteins with different expression patterns were further classified according to protein functions in Table 3. The function of differential proteins mainly participated in carbohydrate metabolism, other stress response and protein modification. In the class A expression pattern, the differential proteins were mainly involved in other stress response, protein modification and reactive oxygen species-related. Under the class B expression pattern, the differential proteins were mainly involved in carbohydrate metabolism, signal transduction, lipid metabolism and amino acid metabolism.

**Table 3:** Functional distribution of differential proteins with different expression patterns

Functions	Number		Total
	A	B	
Protein modification	10	7	17
Protein transport and translation	5	5	10
Signal Transduction	2	10	12
Reactive oxygen species- related	6	2	8
Lipid metabolism	4	8	12
Amino acid metabolism	4	8	12
Carbohydrate metabolism	5	14	19
Other stress response	12	7	19
Total	48	61	109

Note: A. The abundance of protein was continuously down-regulated during the grain substance accumulation; B. The abundance of protein was continuously up-regulated during the grain substance accumulation

### Key differential proteins responsive to stimulus were involved in both response to elevated temperature and grain substance accumulation

**Fig. 6:**
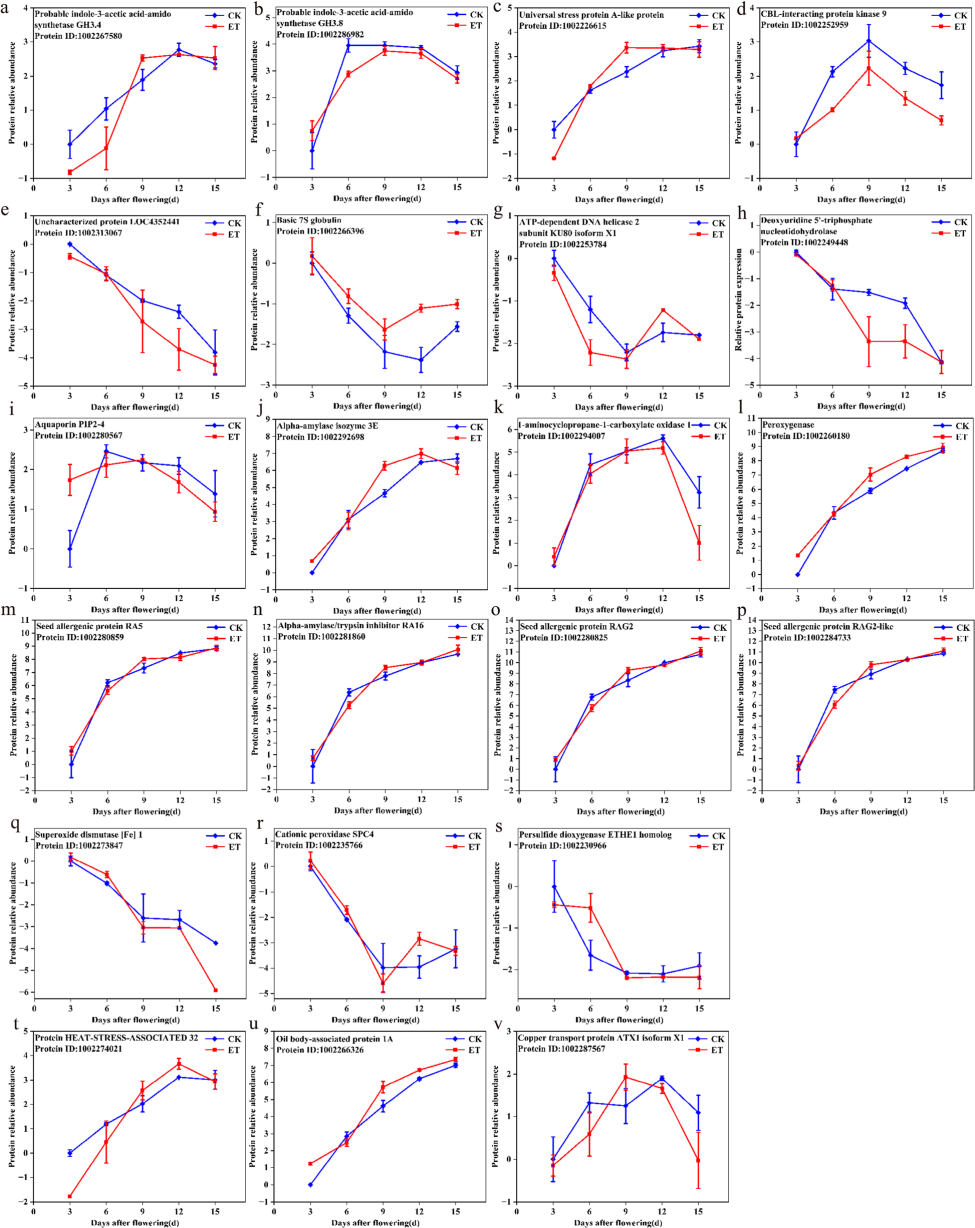
Expression of 22 important proteins which responded to both grain substance accumulation (3-15d after flowering) and elevated temperature (Fold change >  = 2; *P*-value < 0.05). The 22 proteins were divided into 5 categories according to functions: signal transduction, protein modification and transport, storage substance metabolism, reactive oxygen species-related and other stress responses. **a**-**d** The protein functions belonged to signal transduction; **e**–**h** The protein functions belonged to protein transport and modification; **i**-**p** The protein functions belonged to storage substance metabolism; **q**-**s** The protein functions belonged to reactive oxygen species-related; **t**-**v** The protein functions belonged to other stress responses. Note: *CK* Natural temperature, *ET* Elevated temperature. Protein ID was obtained from NCBI

We screened for differential proteins which responded to both grain development (3-15d after flowering) and elevated temperature (Fold change >  = 2; *P*-value < 0.05). Finally, 22 differential proteins were selected for further analysis (Fig. 6). The 22 proteins were divided into 5 categories according to functions: signal transduction, protein modification and transport, storage substance metabolism, reactive oxygen species-related and other stress responses (Table S7).

Probable indole-3-acetic acid-amide synthetase GH3.4 (1,002,267,580), probable indole-3-acetic amide synthetase GH3.8 (1,002,286,982), universal stress protein A-like protein (1,002,226,615) and CBL-interacting protein kinase 9 (1,002,252,959), which involved in signal transduction, gradually increased in protein abundance during grain substance accumulation. Warming decreased the abundance of the four proteins on different days after flowering, especially CBL-interacting protein kinase 9 (1,002,252,959), which decreased by 0.46 and 0.49-folds under elevated temperature at 6d and 15d after flowering, respectively.

Different from the expression pattern of differentially expressed proteins involved in signal transduction, the four proteins involved in protein modification and transport were all down-regulated as grain filling. Under elevated temperature, the abundance of basic 7S globulin (1,002,266,396) was up-regulated from 3d-15d after flowering, especially at 12d after flowering, in which the abundance was significantly up-regulated by 2.42 times. Unknown protein LOC4352441 (1,002,313,067), ATP-dependent DNA helicase 2 subunit KU80 isoform X1 (1,002,253,784) and deoxyuridine 5'-triphosphate nucleotide hydrolase (1,002,249,448) were down-regulated under elevated temperature.

A total of 8 proteins were involved in the storage substance metabolism, and 6 of these proteins were all continuously up-regulated with the grain substance accumulation. The abundance of aquaporin PIP2-4 (1,002,280,567) was significantly up-regulated by 3.34 times at 3d after flowering by elevated temperature. Elevated temperature significantly increased the abundance of Alpha-amylase isoenzyme 3E (1,002,292,698) by 3.02 times at 9d after flowering.

There were three proteins belonged to reactive oxygen species-related, of which the accumulation of superoxide dismutase [Fe] 1 (1,002,273,847) and persulfide dioxygenase ETHE1 homolog (1,002,230,966) was gradually down-regulated during grain substance accumulation. Under elevated temperature, the superoxide dismutase [Fe] 1 (1,002,273,847) was significantly down-regulated to 0.22 times of natural temperature at 15 d after flowering, while cationic peroxidase SPC4 (1,002,235,766) and persulfide dioxygenase ETHE1 homolog (1,002,230,966) were significantly increased by 2.15 and 2.20 times at 12d and 6d after flowering, respectively.

HEAT-STRESS-ASSOCIATED 32 (1,002,274,021), oil body-associated protein 1A (1,002,266,326) and copper transport protein ATX1 isoform X1 (1,002,287,567) were classified as other stress responses protein and gradually increased in relative protein abundance with grain substance accumulation. Elevated temperature resulted in a decreased accumulation of HEAT-STRESS-ASSOCIATED 32 (1,002,274,021) and copper transport protein ATX1 isoform X1 (1,002,287,567) at 3d and 15d after flowering, respectively. However, oil body-associated protein 1A (1,002,266,326) was 2.36-fold and 2.18-fold higher than that under natural temperature at 3d and 9d after flowering.

To verify the reliability of the proteome results, we performed a verification experiment by RT-PCR at the transcriptional level. 10 proteins were selected including the above five functional categories. Except for aquaporin PIP2-4 protein (LOC4343119), the other 9 proteins showed the same tendency in the relative expression level of RT-PCR and DIA proteomic level (Fig. S2).

## Discussion

### The important role of signal transduction and ROS related proteins in grain substance accumulation and response to elevated temperature

**Fig. 7:**
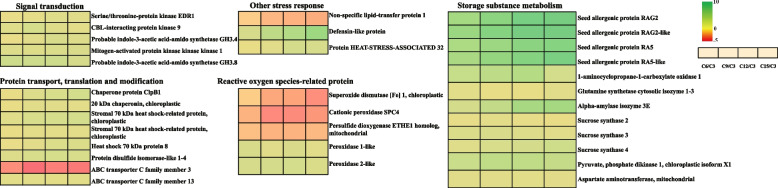
Heatmaps of differentially expressed proteins during grain substance accumulation. According to the functional classification, the important differential proteins were classified as signal transduction, protein transport, translation and modification, storage substance metabolism, reactive oxygen species-related and other stress responses (Fold change >  = 2; *P*-value < 0. 05). Four consecutive squares from left to right indicates different days after flowering (6, 9, 12 and 15d after flowering).Red indicates the abundance of protein was low, and green indicates the abundance of protein was high

To withstand stresses, plants have formatted regulatory pathways and produce various proteins including protein kinases, molecular chaperones, ROS-scavenging proteins, etc. to confer tolerance to stresses [54]. The adverse environment firstly is sensed by plants which further triggers stress-specific signal transduction [55]. Plant hormones are important signal compounds that mediate plant responses to stress [56]. In our study, we identified several signal transduction proteins including probable indole-3-acetic acid-amido synthetase GH3.4 (1,002,267,580) and probable indole-3-acetic acid-amido synthetase GH3.8 (1,002,286,982) which were related to auxin synthesis during grain substance accumulation and response to elevated temperature (Fig. 7). The abundance of probable indole-3-acetic acid-amido synthetase GH3.8 was significantly accumulated at 6d-15d after flowering. However, elevated temperature suppressed the accumulation of probable indole-3-acetic acid-amido synthetase GH3.4 and probable indole-3-acetic acid-amido synthetase GH3.8 which decreased by 0.45-fold and 0.48-fold, respectively. The results showed that the up-regulation of auxin-related proteins may promote grain substance accumulation. Study has indicated auxin synthesis was necessary but not sufficient for heat tolerance [57]. However, elevated temperature inhibited the accumulation of auxin-related proteins. It may be our elevated temperatures are beyond the range that grain can actively regulate.

Protein kinases also play vital roles in signal transduction under stress conditions [58]. It is a central component in plant responses to environmental stresses by adding phosphate groups to control the concentration of ions or molecules which further initiate the corresponding physiological and biochemical reactions to alleviate adversity stress [59, 60]. We identified 3 differential expression protein kinases during grain substance accumulation and 1 differential expression protein kinase under elevated temperature. Under natural temperature, the abundance of serine/threonine-protein kinase EDR1 (1,002,302,490) was up-regulated by 4.44-fold and 3.98-fold at 12d and 15d after flowering compared to 3d. Mitogen-activated protein kinase (MAPK) belongs to serine/threonine-protein kinase and has been validated to join in biotic and abiotic stress responses [61]. Mitogen-activated protein kinase kinase kinase 1 (1,002,252,173) was differentially expressed in grain substance accumulation and the abundance was consistently up-regulated. CBL-interacting protein kinase 9 was the only signal transduction related protein that was differentially expressed during grain substance accumulation and elevated temperature. Calmodulin B-like (CBL) proteins specifically interacted with CBL-Interacting Protein Kinases (CIPKs) to transduce calcium signals [62, 63]. Piao et al. [64] used the loss-of-function mutation of *OsCIPK31* to elucidate *OsCIPK31* is involved in rice germination and seedling growth under abiotic stress. During grain development, CBL-interacting protein kinase 9 obviously accumulated. However, the CBL-interacting protein kinase 9 (1,002,252,959) was down-regulated under elevated temperature especially at 6d-15d after flowering. The decreased expression of signal transduction related proteins under warming was likely to impair the ability of rice grains to respond to elevated temperature.

**Fig. 8:**
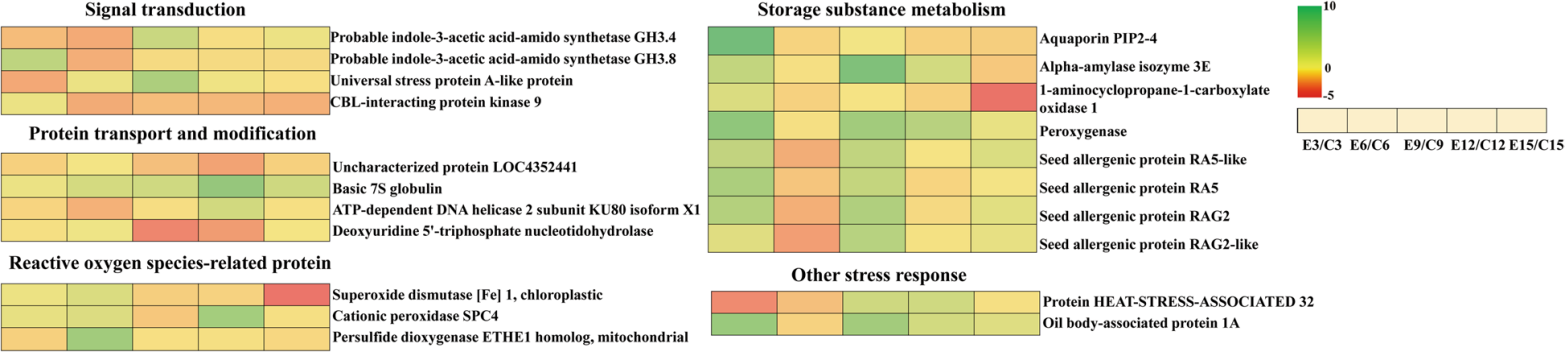
Heatmaps of differentially expressed proteins under elevated temperature. According to the functional classification, the important differential proteins were classified as signal transduction, protein transport and modification, storage substance metabolism, reactive oxygen species-related and other stress responses (Fold change >  = 2; *P*-value < 0. 05). Five consecutive squares from left to right indicates different days after flowering (3, 6, 9, 12 and 15d after flowering) under elevated temperature. Red indicates the abundance of protein was low, and green indicates the abundance of protein was high

Reactive oxygen species (ROS) are considered unavoidable byproducts of the aerobic metabolism of plants [65]. During stress, ROS accumulates significantly in chloroplast, mitochondria, peroxisome and apoplast leading to oxidative damage [66–68]. However, plants have a sophisticated antioxidative mechanism that consists of enzymatic and non-enzymatic components [69, 70]. The superoxide dismutase (SOD) and peroxidase (POD), etc. antioxidant enzymes can regulate the homeostasis of ROS [71]. We identified reactive oxygen species-related proteins including a superoxide dismutase and several peroxidases in grain substance accumulation and response to elevated temperature. The accumulation of major reactive oxygen species-related proteins significantly decreased, such as superoxide dismutase [Fe] 1(1,002,273,847), cationic peroxidase SPC4 (1,002,235,766), and the ETHE1 homologue of persulfide dioxygenase (1,002,230,966) (Fig. 7). However, under elevated temperature, the abundance of superoxide dismutase [Fe] 1(1,002,273,847), cationic peroxidase SPC4 (1,002,235,766) and the ETHE1 homologue of persulfide dioxygenase (1,002,230,966) were improved in response to elevated temperature (Fig. 8). In particular, the cationic peroxidase SPC4 (1,002,235,766) and the ETHE1 homologue of persulfide dioxygenase (1,002,230,966) were 2.15 and 2.20 times higher at 12d and 6d after flowering, respectively. Therefore, rice grains have a strong ability to adapt to elevated temperature by accumulating antioxidants. Differently, superoxide dismutase [Fe] 1 (1,002,273,847) was significantly down-regulated to 0.22 times at 15d after flowering under elevated temperature. It may be that the improvement of enzyme activity is not infinite under stress.

### The accumulation of transport modification related proteins in grain filling and response to elevated temperature

ABC-transporter protein represents one of the largest protein families with transporter activity [72]. It is not only involved in the growth and development of plants, but also responded to abiotic stresses [73]. We identified two ABC transporter proteins which were ABC transporter C family member 3 (1,002,232,580) and ABC transporter C family member 13 (1,002,253,466). Interestingly, the two ABC transporter proteins showed opposite accumulation trends during grain substance accumulation. The accumulation level of ABC transporter C family member 3 (1,002,232,580) decreased with grain substance accumulation. At 15d after flowering, it was only 0.04 times that at 3d after flowering. On the contrary, the accumulation of ABC transporter C family 13 (1,002,253,466) was dramatically up-regulated at 6d-15d after flowering, especially at 15d after flowering.

Maintaining protein homeostasis requires coordination of protein synthesis, folding, quality control, and subcellular targeting [74]. However, elevated temperature disturbed the protein homeostasis resulting in the over-accumulation of unfolded or misfolded proteins in the endoplasmic reticulum [75]. Maintaining the normal folding and promoting the degradation of misfolded proteins are vital for plants to survive under high temperature [76]. Molecular chaperones play a role in maintaining the native conformation of proteins under environmental stress [77]. Many studies have found that overexpressing molecular chaperones of HSP genes can improve tolerance to environmental stress [78, 79]. A total of 17 proteins related to protein modification were identified to be significantly differentially expressed during grain substance accumulation (Table. S6). Among them, 5 chaperone proteins were significantly accumulated during grain substance accumulation. In addition, we also identified differential proteins of protein disulfide isomerase-like 1–4 (1,002,245,724). On the one hand, a protein disulfide isomerase-like enzyme coded by *PDIL1-1* can participate in the maturation of pro-glutelin [80]. On the other hand, it is one of the chaperones in the endoplasmic reticulum (ER) involved in the folding of grain storage proteins [81]. The up-regulated modification-related proteins could stabilize the protein structure and ensure the normal function of proteins to cope with possible external and internal stimuli during grain substance accumulation.

Under elevated temperature, there were 4 differentially expressed proteins related to protein modification and transport. Except for the basic 7S globulin (1,002,266,396), the other three proteins were significantly down-regulated, including the unknown protein LOC4352441 (1,002,313,067). The unknown protein LOC4352441 was significantly down-regulated to 0.4 times at 12d after flowering. Hamzelou et al. [82] identified 10 proteins that improved the abundance in response to drought stress without a characterized function. Therefore, the 10 proteins may be candidates for functional analysis and potential biomarkers of drought resistance for breeders. In our study, the unknown protein LOC4352441 may be helpful to further study the response to elevated temperature in rice grains.

### The accumulation of other stress response proteins in grain filling and response to elevated temperature

During growth and development, plants have acquired a set of overlapping stress response systems to cope with a multitude of adverse environments [83]. During grain substance accumulation, the accumulation of 19 other stress response proteins was changed significantly. For example, non-specific lipid-transfer protein 1(1,002,307,908), defensin-like protein (1,002,249,243) and protein HEAT-STRESS-ASSOCIATED 32(1,002,274,021). Non-specific lipid-transfer proteins play a crucial role in plant growth and development and resistance to biotic and abiotic stress [84]. However, the abundance of non-specific lipid-transfer protein 1 was obviously reduced in our study. Different from non-specific lipid-transfer protein 1, defensin-like protein accumulated continuously. At 12d and 15d after flowering, the content of defensin-like protein was 56.89 and 147.03 folds compared to 3d. Luo et al. [85, 86] showed defensin-like protein coded by *CAL1* and *CAL2* positively regulated Cd accumulation in rice leaves and grains. The protein HEAT-STRESS-ASSOCIATED 32 (1,002,274,021) was the only differential expression protein during grain substance accumulation and response to elevated temperature. Different from the continuous accumulation during grain substance accumulation, the abundance of protein HEAT-STRESS-ASSOCIATED 32 was generally down-regulated under warming. Especially at 3d after flowering, the accumulation under warming was 0.29 times of natural temperature. Lin et al. [87] pointed out HEAT SHOCK PROTEIN101 and HEAT STRESS-ASSOCIATED 32-KD PROTEIN prolonged the effect of heat acclimation in rice seedlings at the posttranscriptional level. We speculated the difference in results may be related to the period, rice part and treatment temperature. The abundance of oil body-associated protein 1A was significantly up-regulated under warming. Studies have shown that oil body-associated protein played an important role in the protection of plant embryos against freeze [88]. Due to the highly complex signal transduction pathways and the interaction of different metabolic components, the response to other stresses under elevated temperature still needs extensive research.

### The accumulation of proteins related to storage substance metabolism in grain substance accumulation and response to elevated temperature

Starch, storage protein and lipid are major storage materials in rice endosperms [49]. A total of 43 differential proteins related to the storage substance metabolism were identified including carbohydrate metabolism, amino acid metabolism and lipid metabolism (Table S6). Pyruvate, phosphate dikinase 1, and chloroplastic isoform X1 (1,002,269,961), sucrose synthase 2 (1,002,277,371), sucrose synthase 3 (1,002,281,018), sucrose synthase 4 (1,002,255,789) and alpha-amylase isozyme 3E (1,002,292,698) were identified to relate to carbohydrate metabolism. Pyruvate phosphate dikinase (PPDK) reversibly interconverts pyruvate, Adenosine triphosphate (ATP), and orthophosphate with phosphoenolpyruvate (PEP), Adenosine monophosphate (AMP), and pyrophosphate [89]. PPDK can regulate gluconeogenesis by providing hexose for starch biosynthesis and provide pyruvate for lipid synthesis [90–92]. The accumulation of pyruvate, phosphate dikinase 1, and chloroplastic isoform X1 (1,002,269,961) increased to 18, 28.64, 29.04, 22.16 folds at 6d, 9d, 12d and 15d after flowering, respectively. Sucrose synthase catalyzes the conversion of sucrose and uridine diphosphate (UDP) into fructose and UDP-glucose, which is an important starting material for storage starch biosynthesis [93–95]. Starch is the most abundant storage substance in the rice endosperm [96]. Therefore, sucrose synthase is vital for grain substance accumulation. Fan et al. [93] demonstrated that overexpression of sucrose synthase coding genes could significantly increase rice starch accumulation in transgenic rice to improve yield and quality. The three sucrose synthases in our study accumulated significantly during grain substance accumulation. In 3d–15d after flowering, the increase in grain weight reflected the accumulation of starch (Fig. 1 B, D). In rice, alpha-amylase isozymes are crucial for the formation of starch granules during grain maturation [97]. However, alpha-amylase may involve in the production of chalky by impairing starch accumulation in the developing endosperm [18]. Under elevated temperature, the abundance of alpha-amylase isozyme 3E (1,002,292,698) was increased by 2.02 folds compared to natural temperature at 9 d after flowering. The up-regulation of alpha-amylase isozyme may be responsible for chalk formation. During grain substance accumulation, glutamine synthetase cytosolic isozyme 1–3 (1,002,249,042) and aspartate aminotransferase (1,002,273,225) were continuously accumulated. Glutamine synthetase catalyzes the assimilation of ammonium into glutamine which is the main way to synthesize nitrogenous organic matter by ammonium [98]. Aspartate aminotransferase, which catalyzes to generate glutamate and oxaloacetate, plays a key role in the metabolic regulation of carbon and nitrogen metabolism [99]. The accumulation of the two proteins may promote the utilization of nitrogen in rice grains, promote the synthesis of amino acids and facilitate the accumulation of proteins (Fig. 2D). In lipid metabolism, we identified some seed allergenic proteins which significantly accumulated during grain filling and response to elevated temperature (Table S6). Zhou et al. [100] found that the overexpression of *RAG2* gene significantly increased grain the content of storage protein and crude lipid, and improved grain chalkiness. During grain substance accumulation, the four seed allergenic protein continuously accumulated. Under elevated temperature, the four seed allergenic proteins accumulated slightly at all times except for a slight decrease in 6d after flowering. The seed allergenic protein RA5 (1,002,280,859) was significant accumulation at 3d after flowering, which may be the key seed allergenic protein to regulate the protein and crude lipid under elevated temperature. The relationship between seed allergenic protein accumulation and grain weight and quality under elevated temperature still needs further study.

Elevated temperature triggers a series of events in plants, involving heat sensing, signal transduction, sophisticated regulatory networks or pathways, and ultimately improved plant physiological and metabolic processes in response to warming [101]. In our study, auxin synthesis and calcium signal associated with signal transduction, protein transport and modification, and reactive oxygen species-related proteins may be key proteins responsive to stimulus under elevated temperature. Plant hormones as signaling compounds are not only involved in the regulation of plant growth and development, but are also able to respond to stress response [102]. In previous studies, auxin signaling can be involved in the plant response to temperature to influence plant growth [103, 104]. In addition, the auxin signaling transduction was also affected by reactive oxygen species which are essential chemical entities and as the key secondary messengers to activate signal transduction to regulate lateral root formation [105, 106]. In our study, the accumulation of probable indole-3-acetic acid-amido synthetase GH3.4 (1,002,267,580) and probable indole-3-acetic acid-amido synthetase GH3.8 (1,002,286,982) which were related to auxin synthesis was decreased, and the abundance of reactive oxygen species-related protein was up-regulated under elevated temperature. We hypothesize that the down-regulation of auxin synthesis-related protein and the increase in reactive oxygen species accumulation under elevated temperature is a strategy for rice to cope with elevated temperature. However, whether reactive oxygen species and auxin synthesis-related proteins are in the same regulatory pathway and jointly involved in specific pathways to regulate rice response to elevated temperature still requires further study. In Protein–protein interaction networks, deoxyuridine 5'-triphosphate nucleotidohydrolase which belongs to protein modification and transport, and superoxide dismutase [Fe] 1, which belongs to reactive oxygen species-related protein, did not have direct interconnections, and the function of proteins with indirect connections remains unknown (Fig. S3). Therefore, in our study, auxin synthesis associated with signal transduction, protein transport and modification, and reactive oxygen species-related proteins may be key proteins responsive to stimulus to further regulate the accumulation of storage substance under elevated temperature. However, the closer interactions among signal transduction, protein transport and modification, and reactive oxygen species-related proteins still need to be further investigated.

## Conclusion

**Fig. 9:**
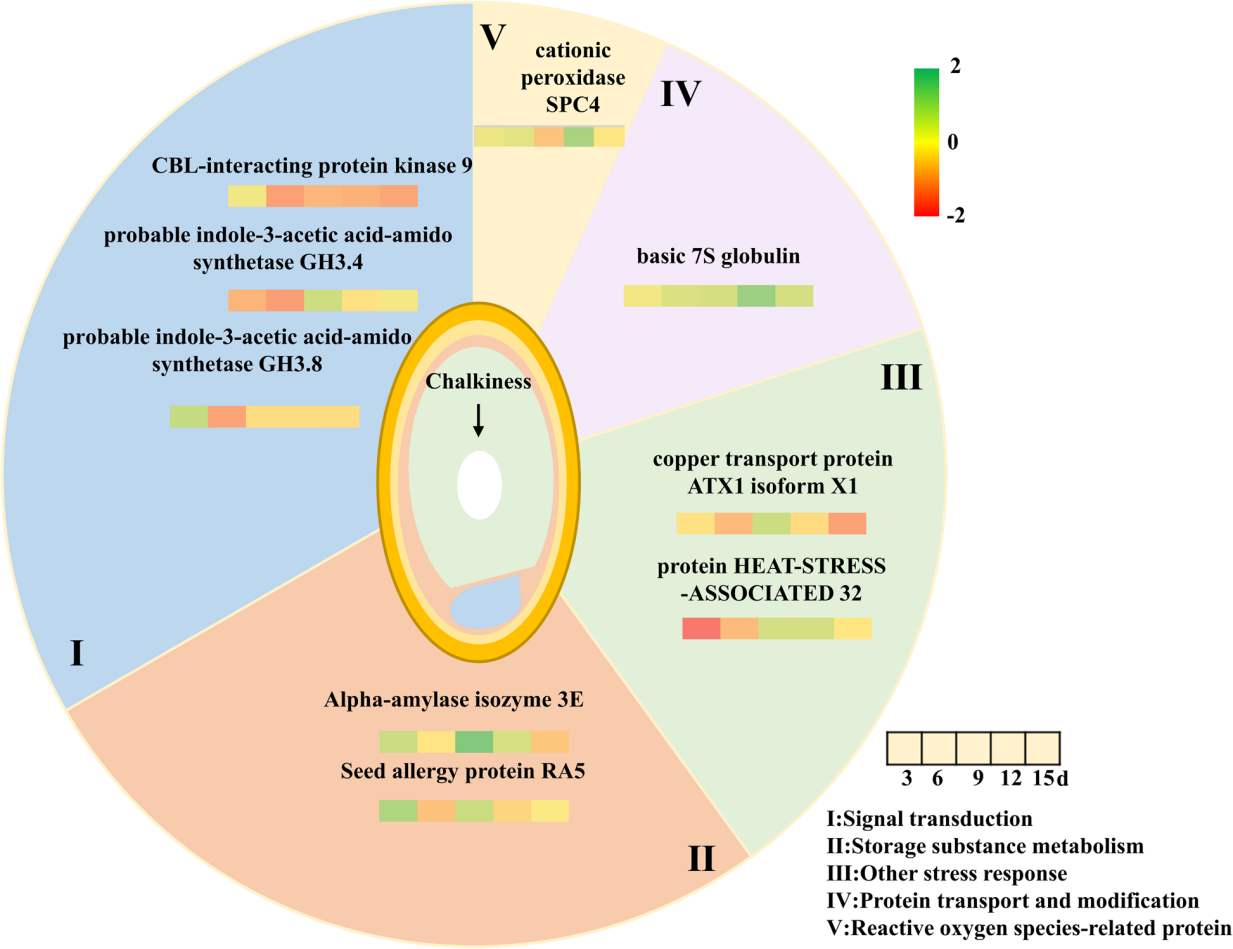
Key proteins responsive to stimulus may regulated grain quality formation under elevated temperature. Five consecutive squares from left to right indicates different days after flowering (3, 6, 9, 12 and 15d after flowering). Values of protein abundance from minimal to maximal are colored from red to green

Elevated temperature affected the accumulation of storage substance and severely deteriorated rice quality, especially significantly increased the chalkiness. We identified some differential expression proteins responsive to stimulus by proteomic and classified into signal transduction, protein transport and modification, storage substance metabolism, reactive oxygen species-related and other stress responses. Under elevated temperature, the abundance of signal transduction and other stress response-related proteins decreased and the accumulation of protein transport and modification and reactive oxygen species-related proteins increased. Together with accumulated alpha-amylase isozyme and seed allergy protein, these proteins regulated the storage substance accumulation and possibly affected rice quality formation (Fig. 9). Among the proteins we identified, the functions of related proteins in grain substance accumulation and response to elevated temperatures still need to be further revealed. The results provide new insights into the formation of rice quality under elevated temperatures and provide a new strategy for quality improvement in rice breeding.

## Materials and methods

### Crop management

The field experiment was carried out in 2020 and 2021 in Danyang City at the experimental base of Nanjing Agricultural University (31^。^56’N, 118^。^59’ E). Wuyujing 3 (W3), was a conventional *japonica* rice variety widely cultivated in the middle and lower reaches of the Yangtze River. W3 was sown on May 26 and May 25 and transplanted on June 13 and June 16 in 2020 and 2021 respectively. Three seedlings per hill were artificially transplanted into fields at 13.3 cm × 30 cm spacing. Nitrogen fertilizer was applied three times (120 kg N ha^−1^ as basal fertilizer, 60 kg N ha^−1^ as tiller fertilizer, and 120 kg N ha^−1^ as panicle fertilizer). Phosphorus (150 kg P ha^−1^ as phosphorus pentoxide) was used as basal fertilizer. Potassium oxide was used as potassium fertilizer with the application rate of 240 kg K ha^−1^ split-applied at basal fertilizer (50%), and panicle fertilizer (50%). Basal fertilizer was manually applied one day before transplanting. Water irrigation and other field management were carried out in accordance with local high-yield cultivation measures. The average temperature and precipitation during grain filling in 2020 and 2021 were shown in Fig. S4.

### Temperature treatment

**Fig. 10:**
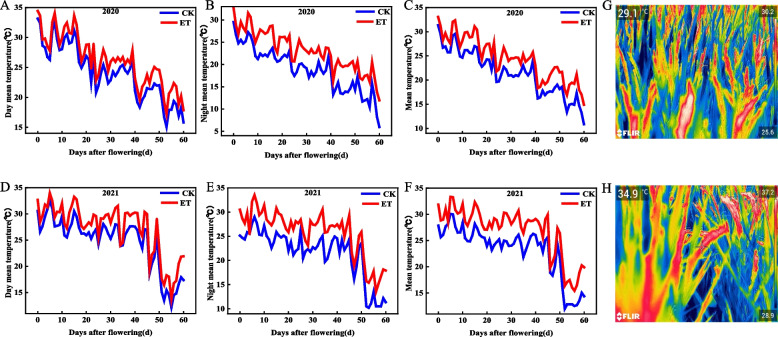
Day mean temperature (**A**,**D**) and Night mean temperature (**B**,**E**) and Mean temperature (**C**,**F**) of rice canopy under natural temperature (CK) and elevated temperature (ET) during grain filling in 2020 and 2021. Thermal images of CK (**G**) and ET (**H**) spikelets at 12d after flowering in the natural state in 2020

This study was a real field temperature experiment with two treatments CK: natural temperature and ET: elevated temperature. The elevated temperature experiment was conducted under actual field warming from the beginning of grain filling to physiological maturity by the Free-Air Temperature Enhancement (FATE). As shown in Fig. S5A, infrared radiation heating was carried out through 12 ceramic infrared heaters (240 V × 1000W) in plots of warming. The ceramic infrared heaters were installed 1.2 m above the rice canopy at an angle of 45° from the horizontal and 30° from the vertical direction. A temperature sensor (HOBO, Onset computer Corp., Bourne, MA, USA) was placed in the middle of the plot to record temperature all day. The effect of elevated temperature was shown in Fig. 10. The mean temperature of rice canopy steadily increased by 3.13 ℃ and 3.24 ℃ in 2020 and 2021 under elevated temperature. The daytime temperature increased by 1.99 ℃ (2020) and 2.41 ℃ (2021), and the nighttime temperature increased by 4.27 ℃ (2020) and 4.08 ℃ (2021) respectively. The effect of elevated temperature on the spikelet temperature was shown in Fig. 10 G and H. The thermal images were photographed between 2:00–3:00 pm at 12 d after flowering using a FLIR ThermaCAMTM S65 system (FLIR Systems Inc., Portland, OR, USA). The experiment was a randomized block design with three replicates.

### Filling rate and yield determination

Panicles were labeled when about one third of spikelets were flowering in 2020 and 2021. The marked panicles were collected at 3, 6, 9, 12, 15, 20, 25, 35 and 45 d after flowering. Samples were put into envelope bags and dried at 80 ℃ to a constant weight in oven. The grain weight after threshing was recorded. According to the grain weight dynamics, the Richards equation was used to fit the grain filling process, and calculated the grain filling rate and key parameters [107].

Ten hills from each replicate plot were sampled at the mature stage to determine yield components in 2020 and 2021. Calculate the number of panicles to determine the panicle. The number of filled spikelets and unfilled spikelets were counted, and the spikelets per panicle and seed setting rate were calculated. The manually separated filled spikelets were dried to constant weight to be weighed and used to calculate the grain weight. Finally, the theoretical yield was calculated by the formula: yield (t ·ha^−1^) = panicles per hectare × spikelets per panicle × grain weight (mg) × seed setting rate × 10^–6^.

### Determination of related indicators of rice quality

Rice grains at maturity stage were collected to determine the rice quality in 2020 and 2021.

Milling quality: 100 g rice grains with three repetitions were shelled by machine JLGJ4.5 of Taizhou Grain Industry instrument Company (Taizhou Grain Industrial Instrument Company, Zhejiang, China). Brown rice rate was obtained by weighing brown rice. Brown rice was milled for 90 s by JNMJ3 rice mill (Taizhou Grain Industry instrument Company, Zhejiang, China) to obtain milled rice. Integrity of more than 80% was manually selected from milled rice to be head rice, and used to calculate the head rice rate.

Appearance quality: The length, width and chalkiness of rice were measured by Scanmaker i800 plus (MICROTEK, Shanghai, China). The thickness of rice grain was measured by a vernier caliper.

Cooking and eating quality: The appearance and taste of rice were determined by the rice taste meter STA1BCN (Satake chemical equipment meg., ltd, Shoukou, Osaka, Japan). Hardness and viscosity of rice were used by hardness viscometer RHS1A (Satake chemical equipment meg., ltd, Shoukou, Osaka, Japan).

Crude protein content: The nitrogen content of brown rice flour was determined by the Dumas combustion method with an NCH analyzer (Sumika Chemical Analysis Service, Tokyo, Japan). The crude protein content = nitrogen content × 5.95 [108].

The amylose starch content was determined according to the people's Republic of China standard GB/17891–1999 high quality rice.

RVA profile: Rice RVA characteristics profile was measured with an RVA (Newport Scientific Pty Ltd., Warriewood, Australia). 3 g rice flour (accounting for 14% moisture basis) was suspended in 25 mL distilled water. The measurement conditions were as follows: 1 min of heating at 50 ℃, 3.8 min of heating from 50 to 95 ℃, maintenance for 2.5 min at 95 ℃, 3.8 min of cooling from 95 ℃ to 50 ℃, and holding for 1.4 min at 50 ℃.

### Protein extraction

Panicles were labeled when about one third of spikelets were flowering. Panicles from the middle and upper primary branch stalks were taken at 3, 6, 9, 12 and 15d after flowering and placed at -80 °C after rapid immersion in liquid nitrogen. The grain was ground into powder for proteomic analysis after stripping the glumes on dry ice in 2018.

Protein extraction was performed as follows: (1) 5% polyvinylpolypyrrolidone (PVPP) powder, an appropriate amount of homogenate buffer and two steel balls were added to the moderate sample. (2) The mixture was shaken with a grinder for 2 min (power = 60 Hz, time = 120 s). Take out the steel ball and add 2 times the volume of saturated phenol Trissaturated Phe, shake well for 15min. (3) Centrifuge at 25,000 g for 20 min at 4 ℃, 5 times the volume of 0.1M cold ammonium acetate/methanol and the final concentration of 10mM DTT was added to the supernatant, mix well and placed in a -20 ℃ refrigerator for 2h. (4) Centrifuge at 25,000 g for 20 min at 4 ℃, discard supernatant, and repeat steps (2), (3) and (4) twice. (5) Add 1mL of cold acetone, put in refrigerator at -20 ℃ for 30min, centrifuge at 25,000 g at 4 ℃ for 20 min to discard supernatant, and repeat this step once. (6) Air-dried precipitate, an appropriate amount of SDS was added, the final concentration of 1XCocktail containing EDTA, mix well, put on the ice for 5min, add final concentration 10mM DTT; (7) Grinder (power = 60HZ, time = 2min) was used to break and split, and the supernatant was discarded after centrifugation at 25,000 g for 15 min at 4 ℃. Add final thick DTT with a degree of 10mM was placed in a 56 ℃ water bath for 1h. (8) Add IAM with a final concentration of 55mM and placed it in a dark room for 45min. (9) Add 1mL cold acetone, place in -20 ℃ refrigerator for 2h, centrifuge at 25,000 g for 15 min at 4 ℃ to discard the supernatant. (10) Air-dried precipitate, add an appropriate amount of lysis buffer without SDS to the precipitate of each tube, using a grinder (power = 60HZ, time = 2 min) to break and crack. (11) The supernatant was proteins used for quantification which was obtained at 25,000 g for 15 min at 4 ℃.

### Protein extraction quality control

Bradford assay was used to measure the concentration of protein [109]. The quality of the samples was evaluated by SDS-PAGE.

### Protein enzymatic hydrolysis

(1) Each sample (100 μg) was digested with Trypsin enzyme (2.5 μg) at 37 °C for 4 h. (2) Trypsin enzyme (2.5 μg) was added again and digestion was continued for 8 h at 37 °C. (3) The enzymatic peptides were desalted and vacuumed to dryness.

### High pH RP separation

Mix 10ug of all samples, and 200 ug mixture was diluted with 2 mL of mobile phase A (5% ACN pH 9.8) and injected into the Shimadzu LC-20AB HPLC system coupled with a Gemini high pH C18 column (5 um, 4.6 × 250 mm). The sample was eluted at a flow rate of 1 mL/min by gradient: 5% mobile phase B (95% CAN, pH 9.8) for 10 min, 5% to 35% mobile phase B for 40 min, 35% to 95% mobile phase B for 1 min, flow Phase B lasted 3 min and 5% mobile phase B equilibrated for 10 min. Monitor the elution peak at a wavelength of 214 nm and collected the component every minute. Components were combined into 10 fractions to freeze-dried.

### DDA (Data Dependent Acquisition) and DIA (Data Independent Acquisition) analysis by nano-LC–MS/MS

The freeze-dried peptide was redissolved with mobile phase A (2% ACN, 0.1% FA), centrifuged at 20,000 g for 10 min, and the supernatant was injected into Ultimate 3000 UHPLC (Thermo Scientific, Waltham, MA, USA) to separate. Samples were first enriched and desalted in trap columns, followed by connection to a self-packing C18 column (150 um inner diameter, 1.8 um column material particle size, approximately 35 cm column length), separated by different gradients at 300 nL/min flow rate: 5% mobile phase B (98% ACN, 0.1% FA) for 0–5 min; mobile phase B increased linearly from 5 to 25% for 5–120 min; mobile phase B increased from 25 to 35% for 120–160 min; mobile phase B increased from 35 to 80% for 160–170 min; 80% mobile phase B for 170–175 min; 5% Mobile Phase B for 175–180 min. The nanoliter liquid phase separation end was directly connected to the mass spectrometer.

The Q Exactive HF mass spectrometer (Thermo Fisher Scientific, San Jose, CA, USA) was applied to DDA mode detection. Main parameters: ion source voltage was set to 1.6 kV; MS scan range was 350–1500 m/z; MS resolution was set to 120,000; Maximal injection time (MIT) was set to 50 ms; MS/MS collision type was set to higher-energy collisional dissociation (HCD), normalized collision energy (NCE) was set to 28; MS/MS resolution was set to 30,000; MIT was set to 100 ms, and dynamic exclusion time was set to 30 s. The starting m/z for MS/MS was fixed to 100; the screening conditions of the precursor for MS/MS scan were: charge 2 + to 7 + , top 30 precursors with intensity over 20,000. The automatic gain control (AGC) was set to 3e6 for MS and 1e5 for MS/MS.

For DIA analysis, the same nano-LC system and gradient were used as DDA analysis. The DIA MS parameters were set as below: MS scan range was 400–1250 m/z; MS resolution was set to 120,000; MIT was set to 50 ms; DIA isolation window was set to 17 m/z with loop count 50; MIT was set automatic; scanned at resolution 30,000; stepped NCE: 22.5, 25, 27.5; The target of AGC was 1e6.

### Bioinformatic analysis

DDA data was identified within MaxQuant 1.5.3.30 integrated by Andromeda search engine [110]. Spectronaut™ was used for identification results for spectral library construction [111]. For DIA data, the deconvolution and extraction were by the constructed spectral library information, and the quality control was completed by mProphet algorithm. Differential protein screening satisfied the fold change >  = 2 and *P* value < 0.05 and further performed GO and KEGG pathway analysis.

### RT-PCR (real-time reverse transcription polymerase chain reaction) Analysis

10 key differential proteins were screened for RT-PCR experiments to validate the reliability of proteomics in 2021. Total RNAs were extracted from shelled grains by the RNAprep Pure Plant Kit (TIANGEN, Beijing, China). Analyzing concentration and purity of total RNA and identifying high-quality RNAs were used for reverse transcription using Takara’s PrimeScriptTM RT Kit (Takara Biotechnology, Tokyo, Japan). RT-PCR analysis was conducted by the Biosystems 7300 and StepOnePlusTM real-time PCR system according to Wang et al. [52]. Actin and Ubq were used as reference genes. Primers were shown in Table S8.

### Data analysis

Microsoft Excel 2019 was used for data integration (Microsoft Corporation, WA, USA). ANOVA analysis was carried out by SPSS 26 software (SPSS Inc., Chicago, IL, USA). A least significant difference (LSD) of 0.05 was considered significant differences among different treatments. Origin 8.1 (OriginLab Corporation, MA, USA) was used to draw figures.

### Supplementary Information

**Additional file 1: Supplementary table S1-S8. **

**Additional file 2: Supplementary figure S1-S5. **

## Data Availability

All data supporting the findings of this study are available within the paper and its supplementary data is published online.

## Abbreviations

2-DE: Two-dimensional electrophoresis
HSP: Heat shock proteins
DIA: Data Independent Acquisition
PCA: Principal Component Analysis
GO: Gene Ontology
KEGG: Kyoto encyclopedia of genes and genomes
ABA: Abscisic acid
BRs: Brassinosteroids
CK: Cytokinin
SA: Salicylic acid
MAPK: Mitogen-activated protein kinase
MAPKK: Mitogen-activated protein kinase kinase
CBL: Calmodulin B-like
CIPKs: CBL-Interacting Protein Kinases
CIPKs: CBL-Interacting Protein Kinases
CIPK: CBL Interacting Protein Kinase
ROS: Reactive oxygen species
SOD: Superoxide dismutase
CAT: Catalase
UDP: Uridine diphosphate
PPDK: Pyruvate phosphate dikinase
ATP: Adenosine triphosphate
PEP: Phosphoenolpyruvate
AMP: Adenosine monophosphate
RA: Rice seed allergy protein
FATE: Free-Air Temperature Enhancement
RVA: Rapid viscosity analyzer
DDA: Data Dependent Acquisition
RT-PCR: Real-time reverse transcription polymerase chain reaction
