# Supplementary material for: Stimulus-responsive proteins involved in multi-process regulation of storage substance accumulation during rice grain filling under elevated temperature
Source: BMC Plant Biol. 2023 Nov 8;23:547. doi: 10.1186/s12870-023-04563-7 (PMC10631114; doi:10.1186/s12870-023-04563-7)
Supplement: Supplementary file 2 — Additional file 2: Supplementary figure S1-S5. [file 12870_2023_4563_MOESM2_ESM.pdf]

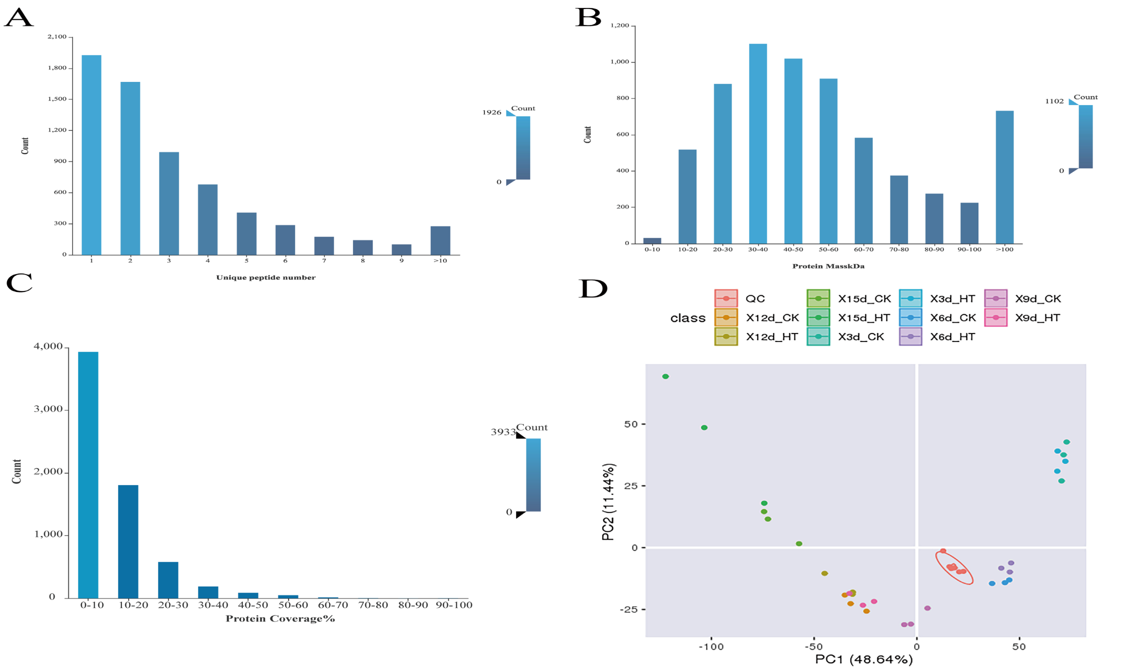

Fig. S1 Basic information statistic of data dependent acquisition (DAA) and principal component analysis. **A** Unique peptide number distribution. **B** Proteins mass distribution. **C** Distribution of protein coverage. **D** Principal component analysis. Note: HT represents elevated temperature and has the same meaning with ET.

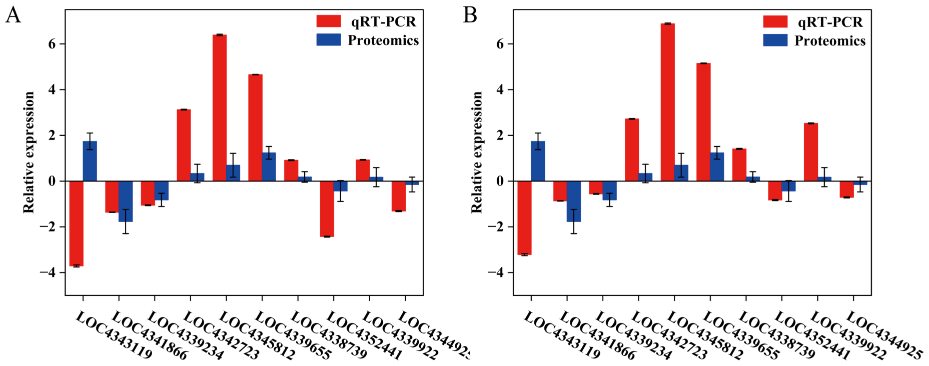

Fig. S2 Validation of proteomics results. A and B use different reference genes respectively. **A** Actin; **B** Ubq. The x-axis represents selected gene symbol, y-axis represents the log2-fold change of relative expression level of RT-PCR (red) and DIA proteomic data (blue) under elevated temperature compared to natural temperature at 3d after flowering. Negative values of expression levels mean that these genes and proteins are down-regulated under elevated temperature. Bars are the standard error.

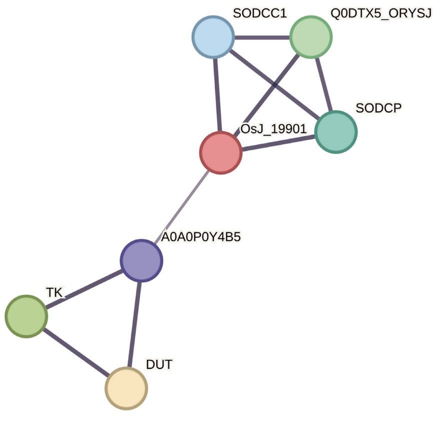

Fig. S3 Protein–protein interaction networks associated with deoxyuridine 5’-triphosphate nucleotidohydrolase and superoxide dismutase [Fe] 1. DUT: Deoxyuridine 5'-triphosphate nucleotidohydrolase; TK: Thymidine kinase; A0A0P0Y4B5: Os11g0615250 protein; OsJ_19901: Superoxide dismutase [Fe] 1, chloroplastic; SODCC1: Superoxide dismutase [Cu-Zn] 1; SODCP: Superoxide dismutase [Cu-Zn]; Q0DTX5_ORYSJ: Os03g0219200 protein. The line thickness indicates the strength of data support of the protein interaction relationship.

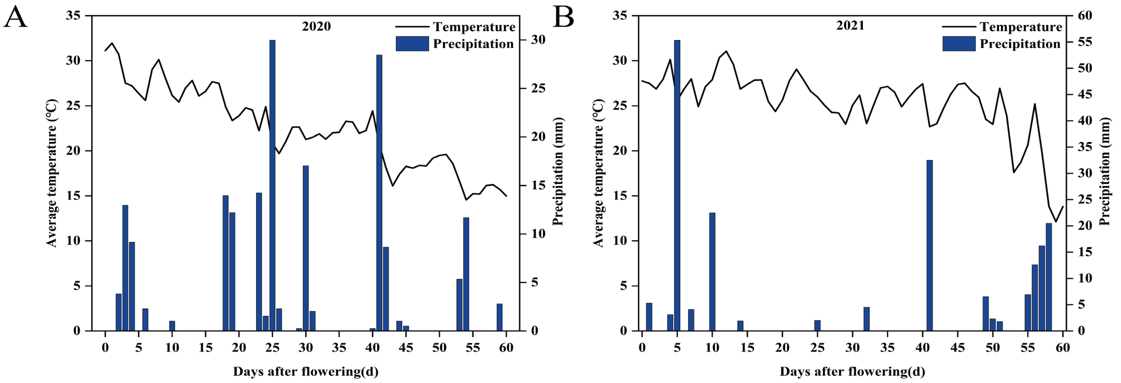

Fig. S4 The temperature and precipitation at the experimental base of Nanjing Agricultural University during grain filling in 2020 (**A**) and 2021 (**B**).

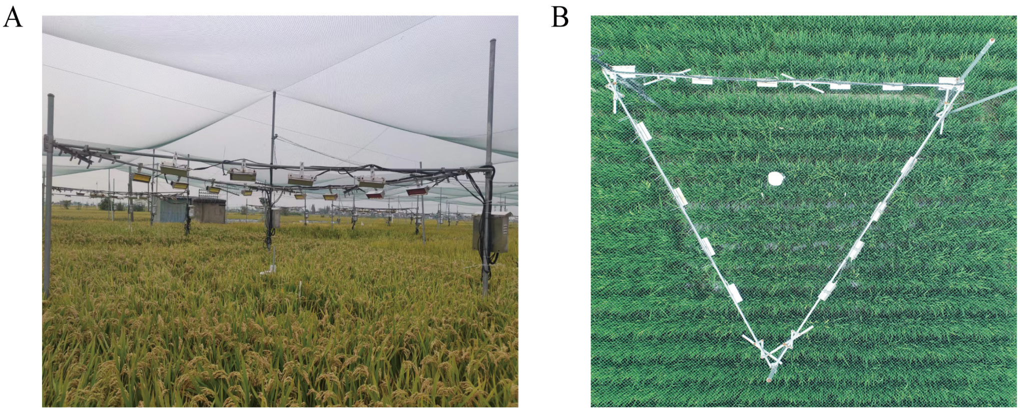

Fig. S5 Actual field warming scene. **A** FATE facility for warming plot. **B** Aerial view of the warming plots.
